# Elevated CDK5R1 predicts worse prognosis in hepatocellular carcinoma based on TCGA data

**DOI:** 10.1042/BSR20203594

**Published:** 2021-01-07

**Authors:** Zhili Zeng, Zebiao Cao, Enxin Zhang, Haifu Huang, Ying Tang

**Affiliations:** 1 The First School of Clinical Medicine, Guangzhou University of Chinese Medicine, Guangzhou, Guangdong 510405, China; 2 Department of Oncology, Shenzhen Hospital (Futian) of Guangzhou University of Chinese Medicine, Shenzhen, Guangdong 518000, China; 3 Department of Oncology, The First Affiliated Hospital of Guangzhou University of Chinese Medicine, Guangzhou, Guangdong 510405, China; 4 Department of Oncology, Lingnan Medical Research Center of Guangzhou University of Chinese Medicine, Guangzhou, Guangdong 510405, China

**Keywords:** CDK5R1, GSEA, Hepatocellular carcinoma, Prognostic value, TCGA

## Abstract

**Background:** Hepatocellular carcinoma (HCC) is a malignant tumor with rapid progression, high recurrence rate and poor prognosis. The objective of our investigation was to explore the prognostic value of CDK5R1 in HCC.

**Methods:** The raw data of HCC raw data were downloaded from The Cancer Genome Atlas (TCGA) database. The Wilcoxon signed-rank test, Kruskal–Wallis test and logistic regression were applied to investigate the relevance between the CDK5R1 expression and clinicopathologic characteristics in HCC. Kaplan–Meier and Cox regression analysis were employed to examine the association between clinicopathologic features and survival. Gene set enrichment analysis (GSEA) was applied to annotate the biological function of CDK5R1.

**Results:** CDK5R1 was highly expressed in HCC tissues. The high expression of CDK5R1 in HCC tissues was significantly associated with tumor status (*P*=0.00), new tumor event (*P*=0.00), clinical stage (*P*=0.00) and topography (*P*=0.00). Elevated CDK5R1 had significant correlation with worse overall survival (OS; *P*=7.414e−04), disease-specific survival (DSS; *P*=5.642e−04), disease-free interval (DFI; *P*=1.785e−05) and progression-free interval (PFI; *P*=2.512e−06). Besides, univariate and multivariate Cox regression analysis uncovered that increased CDK5R1 can independently predict adverse OS (*P*=0.037, hazard ratio [HR]= 1.7 (95% CI [1.0–2.7])), DFI (*P*=0.007, hazard ratio [HR]= 3.0 (95% CI [1.4–6.7])), PFI (*P*=0.007, hazard ratio [HR]= 2.8 (95% CI [1.3–5.9])). GSEA disclosed that notch signaling pathway and non-small cell lung cancer were prominently enriched in CDK5R1 high expression phenotype.

**Conclusions:** Increased CDK5R1 may act as a promising independent prognostic factor of poor survival in HCC.

## Introduction

Primary liver cancer ranks as the fourth most common malignant tumor and the sixth leading cause of cancer incidence in the world, with a 5-year survival rate of 18% [1]. Hepatocellular carcinoma (HCC) constitutes 85–90% of primary liver cancer [2], we mainly focus on HCC in the present study. Although local hepatectomy makes it possible to cure HCC, the overall survival outcome of HCC remains poor. The 5-year local recurrence rate after radical resection is much more than 70% [3]. When HCC related symptoms occur, the average survival time of patients is just approximately 3–4 weeks [4]. Take into account this situation, early prediction of the prognosis before and after treatment is of great significance to improve the 5-year survival rate. On the one hand, it is the key step for the doctor to formulate the correct treatment plan [5]; on the other hand, it is helpful to encourage patients to actively strengthen the monitoring of abnormal indicators, detect abnormalities in time, and treat as early as possible. However, a robust prognostic biomarker of HCC remains limited.

Cyclin-dependent kinase 5 (CDK5) is a unique member of the cyclin-dependent kinases (Cdks) family of serine/ threonine kinases [6]. CDK5 not only plays an important regulatory role in the physiological and pathological processes of the nervous system, but also regulates cell apoptosis and senescence, and works in a variety of tumors [7–9]. Recent studies have found that CDK5 has the effect of driving G1-S and RB phosphorylation in medullary thyroid carcinoma models [10]. It must bind to the activator to exert its activity. P35 is one of the two activators of CDK5, which is encoded by Cyclin-dependent kinase 5 regulatory subunit 1 (CDK5R1), and thus CDK5R1 plays a crucial role in the proper activity of CDK5 [8]. Previous studies have reported that overexpressed CDK5 and CKD5R1 (P35) could promote the progression and metastasis of lung cancer [11], similar results can be seen in melanoma [12], pancreatic cancer [13], large B-cell lymphoma [14] and head and neck squamous cell carcinoma [15]. However, the role and clinical significance of CDK5 and CKD5R1 (P35) in hepatocellular carcinoma have not been reported so far. This article seeks to explore the role of CDK5R1 in HCC and its potential prognostic value.

## Materials and methods

### Patient information

The RNA-sequencing data and corresponding patient clinical information were collected from the TCGA data repository (https://portal.gdc.cancer.gov/repository), involving 374 HCC samples and 50 normal samples, and workflow type was HTSeq-FPKM. The clinical features of HCC patients including age, serum AFP value, BMI, family history, clinical stage, topography (T), lymph node (N), metastasis (M), residual tumor, tumor status, gender, vascular invasion, histologic grade, Child-Pugh, new tumor event, virus, tumor weight, risk factor (alcohol consumption and/or viral hepatitis), postoperative ablation embolization and radiation were recorded. Some unavailable or unclear clinical information was removed. Moreover, in order to verify the expression of CDK5R1 in HCC tissues, gene expression profiles of GSE121248 and GSE62232 were downloaded from the Gene Expression Omnibus (GEO) database. The selection criteria for the data set were: (1) primary hepatocellular carcinoma; (2) complete microarray data; (3) containing cancerous and matched paracancerous tissues (4) the cause of HCC has a wide coverage, including viral infections such as HCV and HBV, heavy drinking, non-alcoholic steatohepatitis and so on.

### Enrichment analysis of GSEA

GSEA is a method that can be used for analysis and calculations so as to ascertain whether the apriori defined group of genes has a consistent and statistically significant difference between two biologic status [16]. In the present study, an ordered list of all genes was firstly produced based on the basis of their association with CDK5R1 expression by GSEA. The expression level of CDK5R1 was served as a phenotype label. The number of gene set permutations were 1000 times for each analysis. The statistical significance of pathways is dependent on normal *P*-value <0.05 and false discovery rate (FDR) *q*-val<0.05.

### Statistical analysis

All statistical analyses were performed with R (version 3.6.1, 2019-07-05, R Foundation, Vienna, Austria), the expression of CDK5R1 between HCC and normal groups was compared by Wilcoxon rank sum tests, and adjacent normal tissues by Wilcoxon signed-rank tests. The relationship between CDK5R1 expression and clinicopathologic characteristics were conducted on the Wilcoxon signed-rank test or Kruskal–Wallis test and logistic regression. The association between the expression of CDK5R1 and survival outcome along with other clinicopathological characteristics was carried out using Cox regression analysis and Kaplan–Meier. In the Cox regression analysis, *P*<0.05 means statistically significant. The median expression value of CDK5R1 was considered to be the cut-off value.

### Construction of PPI network

To investigate the interaction between CDK5R1 and other genes, we established a CDK5R1-related PPI network via the Search Tool for the Retrieval of Interacting Genes/Proteins (STRING) database (https://string-db.org/) [17] with a minimum required interaction score >0.4, and Cytoscape 3.7.1 [18] was applied to visualize these interactions after hiding the disconnected nodes.

## Results

### Clinicopathologic features of patients with HCC

Clinicopathologic features of patients with HCC including age, serum AFP value, BMI, family history, stage, topography (T), lymph node (N), metastasis (M), residual tumor, tumor status, gender, vascular invasion, histologic grade, Child-Pugh, new tumor event, virus, tumor weight, risk factor (alcohol consumption and/or viral hepatitis), postoperative ablation embolization and radiation were downloaded from TCGA database (Table 1). A total of 121 female and 250 male patients were involved in the present study, 90.8% (*n*=336) of them were over age 40. There were 158 of 335 (47.2%) overweight patients, whose BMI were more than 25. Most of patients (65%, *n*=208) didn’t have family history. Moreover, most patients (65.6%, *n*=235) had risk factors, such as alcohol consumption and/or viral hepatitis. The tumor grade included 232 (63.4%) G1-G2 and 134 (36.6%) G3-G4. The stage I-II was found in 257 (74.1%) patients and stage III-IV in 90 (25.9%). Tumor status involved 201(57.1%) tumor free and 151(42.9%) with tumor. The topography included 74.7% (*n*=275) T1-T2 and 25.3% (*n*=93). A total of 4 of 256 (1.6%) patients had lymph node metastasis, 4 of 270 (1.5%) patients had distant metastases, 109 of 315 (34.6%) cases had vascular invasion and 169 patients had new tumor event after treatment. As for Child-Pugh, most of the cases (90.8%, *n*=217) were Child-Pugh A. Besides, 22 (9.2%) cases were Child-Pugh B-C. Serum AFP value<20 was found in 147(52.9%) cases, 20≤ AFP<400 in 66 (23.7%) and AFP ≥400 in 65 (23.4%). The weight of the tumors removed exceeded 500 grams in 248 (83.2%) cases, 500< *W* ≤1000 in 30 (10.1%) cases, *W* > 1000 in 20 (6.7%) cases. Besides, 2.3% (8 of 346) patients had undergone radiation therapy and 8.1% (28 of 347) cases had undergone postoperative ablation embolization.

**Table 1:** HCC patient characteristics based on TCGA

Clinical characteristics	Total	%
Age (years)		
>40	336	90.8
≤40	34	9.2
Gender		
male	250	67.4
female	121	32.6
BMI		
≥25	158	47.2
<25	177	52.8
Family history		
Yes	112	35.0
No	208	65.0
Histologic grade		
G1-G2	232	63.4
G3-G4	134	36.6
Clinical stage		
I-II	257	74.1
III-IV	90	25.9
T		
T1-T2	275	74.7
T3-T4	93	25.3
N		
N0	252	98.4
N1	4	1.6
M		
M0	266	98.5
M1	4	1.5
Residual tumor		
R0	324	94.7
R1	18	5.3
Tumor status		
tumor free	201	57.1
with tumor	151	42.9
Vascular invasion		
Yes	109	34.6
No	206	65.4
Child-Pugh		
A	217	90.8
B-C	22	9.2
AFP		
AFP<20	147	52.9
20<AFP<400	66	23.7
AFP≥400	65	23.4
New tumor event		
Yes	169	48.3
No	181	51.7
Risk factor		
Alcohol consumption and viral hepatitis	39	11.0
Alcohol consumption	79	22.1
Viral hepatitis	117	32.7
Neither	123	34.4
Postoperative ablation embolization		
Yes	28	8.1
No	319	91.9
Radiation therapy		
Yes	8	2.3
No	338	97.7

AFP = alpha fetal protein; BMI = Body Mass Index; M = distant metastasis; N = lymph node metastasis; T = topography distribution.

### CDK5R1 was overexpressed in HCC

**Figure 1:**
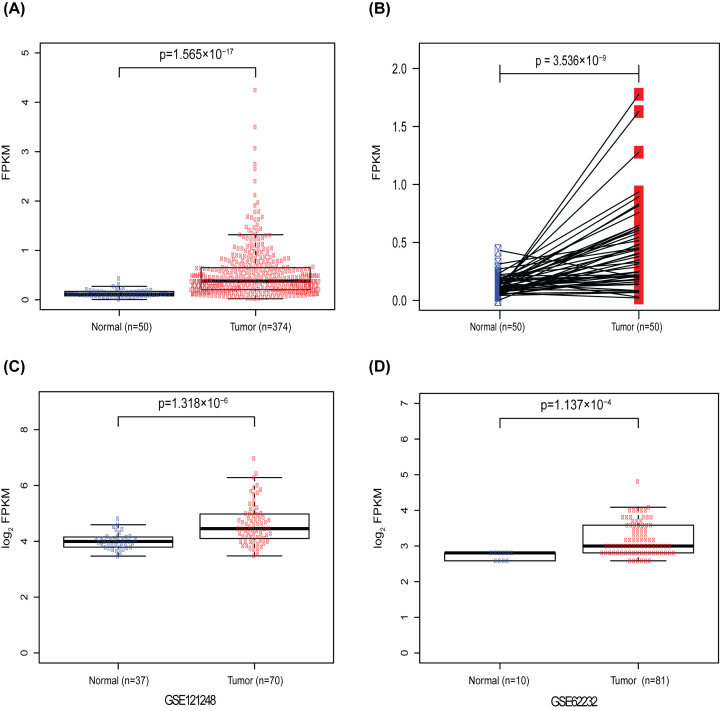
CDK5R1 is elevated in HCC (**A**) CDK5R1 showed prominently high expression in HCC samples than in normal samples via Wilcoxon rank sum test. (**B**) The expression of CDK5R1 was significantly increased in HCC tissues compared with adjacent non-cancerous tissues via Wilcoxon singed-rank test. (**C** and** D**) showed CDK5R1 was prominently elevated in HCC samples from GSE 121248 and GSE 62232; CDK5R1, Cyclin-dependent kinase 5 regulatory subunit 1.

In our research, Wilcoxon rank sum test was used to compare the CDK5R1 expression in 374 HCC tissues and 50 normal tissues. CDK5R1 was significantly elevated in HCC (*P*=1.565e−17) (Figure 1A). In addition, compared with 50 adjacent normal tissues, the expression of CDK5R1 was prominently increased in HCC (*P*=3.536e−09) based on Wilcoxon signed-rank tests (Figure 1B). Further, to verify CDK5R1 expression in other datasets, we downloaded GSE 121248 and GSE 62232 datasets from GEO database. The results also indicated that the expression of CDK5R1 was high in HCC compared with normal tissues (Figure 1C,D).

### The effects of overexpressed CDK5R1 on clinicopathological characteristics

**Figure 2:**
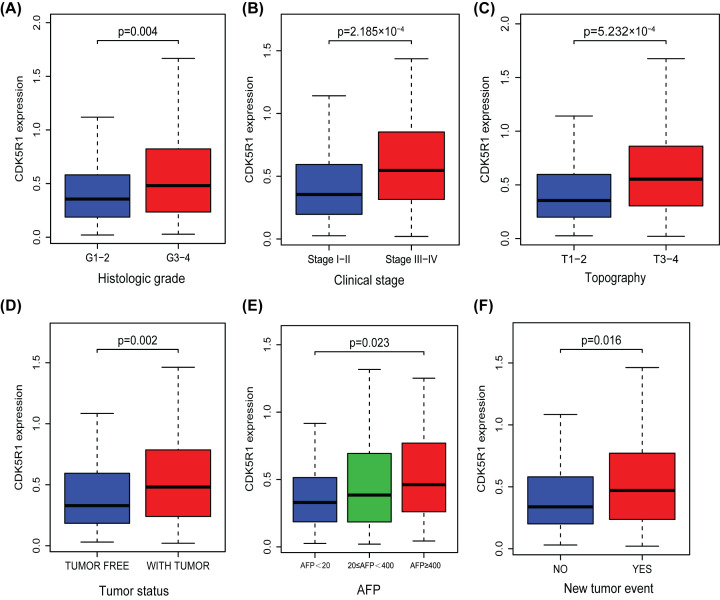
Association between CDK5R1 expression and clinicopathologic characteristics As we can see from panels (**A**–**F**), elevated CDK5R1 was significantly correlated with (A) histologic grade, (B) clinical stage, (C) topography, (D) tumor status, (E) AFP, (F) New tumor event; AFP, alpha fetal protein; M, distant metastasis; N, lymph node metastasis; T, topography distribution.

As shown in (Figure 2A–F), increased CDK5R1 had a significant correlation with histologic grade ((G1-2 vs. G3-4, *P*=0.004), clinical stage (Stage I−II vs. Stage III−IV, *P*=2.185e−04), topography (T1-2 vs. T3-4, *P*=5.232e−04), tumor status (*P*=0.002), AFP (AFP<20 vs. 20≤AFP<400 vs. AFP≥400, *P*=0.023) and new tumor event (*P*=0.016).

Logistic regression was applied to analyze the relationship between CDK5R1 expression and clinicopathologic features (Table 2). We found that overexpressed CDK5R1 was significantly associated with tumor status (OR = 2.28 for with tumor vs. tumor free, *P*=0.00), new tumor event (OR = 1.95 for yes vs. no, *P*=0.00), clinical stage (OR = 2.10 for III-IV vs. I-II, *P*=0.00) and topography (OR = 2.08 for T3-4 vs. T1-2, *P*=0.00). Taken together, high expression of CDK5R1 (based on median expression value) was closely related to worse clinicopathologic characteristics and prone to have a poor prognosis.

**Table 2:** Relationship between CDK5R1 expression and clinicopathologic figures by logistic regression

Clinical characteristics	Total (*N*)	Odds ratio in CDK5R1 expression	*P*-value
Age (>40 vs. ≤40)	370	1.00 (0.49–2.04)	1.000
Gender (male vs. female)	371	0.88 (0.57–1.35)	0.560
BMI (≥25 vs. <25)	335	0.76 (0.49–1.17)	0.210
Family history (yes vs. no)	320	0. 9 (0.57–1.42)	0.640
Child-Pugh (B-C vs. A)	239	1.23 (0.51–3.04)	0.640
AFP			
AFP≥400 vs. AFP<20	212	1.68 (0.93–3.05)	0.080
20≤AFP<400 vs. AFP<20	213	1.27 (0.71–2.28)	0.420
AFP≥400 vs. 20≤AFP<400	131	1.32 (0.67–2.65)	0.420
Tumor status (with tumor vs. tumor free)	352	2.28 (1.48–3.52)	0.000
Tumor weight			
*W* > 1000 vs. *W* ≤ 500	268	1.05 (0.42–2.64)	0.920
1000 ≥ *W* > 500 vs. *W* ≤ 500	278	1.57 (0.73–3.49)	0.250
*W* > 1000 vs. 1000 ≥ *W* > 500	50	0.67 (0.21–2.09)	0.490
Vascular invasion (yes vs. no)	315	1.23 (0.77–2.00)	0.380
New tumor event (yes vs. no)	350	1.95 (1.28–3.00)	0.000
Residual tumor (R1-2 vs. R0)	342	1.00 (0.38–2.62)	1.000
Histologic grade (G3-4 vs. G1-2)	366	1.53 (1.00–2.35)	0.050
Clinical stage (III-IV vs. I-II)	347	2.10 (1.29–3.47)	0.000
T (T3-4 vs. T1-2)	368	2.08 (1.29–3.40)	0.000
N (N1 vs. N0)	256	1.00 (0.12–8.44)	1.000
M (M1 vs. M0)	270	0.33 (0.02–2.60)	0.340
Radiation therapy (yes vs. no)	352	1.00 (0.23–4.29)	1.000
Postoperative ablation embolization (yes vs. no)	353	1.01 (0.46–2.20)	0.987
Risk factor			
Viral hepatitis versus neither	240	1.21 (0.72–2.02)	0.467
Alcohol consumption versus neither	202	1.16 (0.66–2.06)	0.600
Alcohol consumption and viral hepatitis versus neither	162	0.66 (0.31–1.36)	0.264
Alcohol consumption versus viral hepatitis	196	0.96 (0.54–1.72)	0.897
Alcohol consumption and viral hepatitis versus viral hepatitis	156	0.54 (0.25–1.13)	0.107
Alcohol consumption and viral hepatitis versus alcohol consumption	118	0.56 (0.25–1.22)	0.152

Abbreviations: AFP, alpha fetal protein; BMI, body mass index; CDK5R1, cyclin-dependent kinase 5 regulatory subunit 1; M, distant metastasis; N, lymph node metastasis; T, topography distribution.

### Correlation between clinicopathologic features and survival

**Figure 3:**
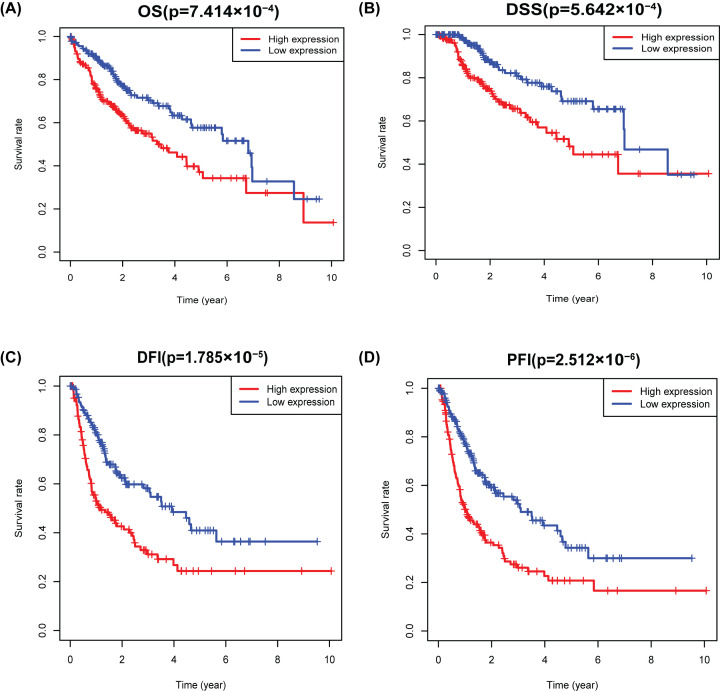
Survival outcomes based on Kaplan–Meier analysis Kaplan–Meier survival analysis showed that increased CDK5R1 was prominently associated with poor (**A**) OS, (**B**) DSS, (**C**) DFI, (**D**) PFI; DFI, disease-free interval; DSS, disease-specific disease; OS, overall survival; PFI, progression-free interval.

Kaplan–Meier unclosed that elevated CDK5R1 had a significant correlation with worse overall survival (OS; *P*=7.414e−04), disease-specific survival (DSS; *P*=5.642e−04), disease-free interval (DFI; *P*=1.785e−05) and progression-free interval (PFI; *P*=2.512e−06), which suggested that HCC patients with high CDK5R1 had a tendency to have shorter survival time than that with low CDK5R1 (Figure 3A–D).

**Figure 4:**
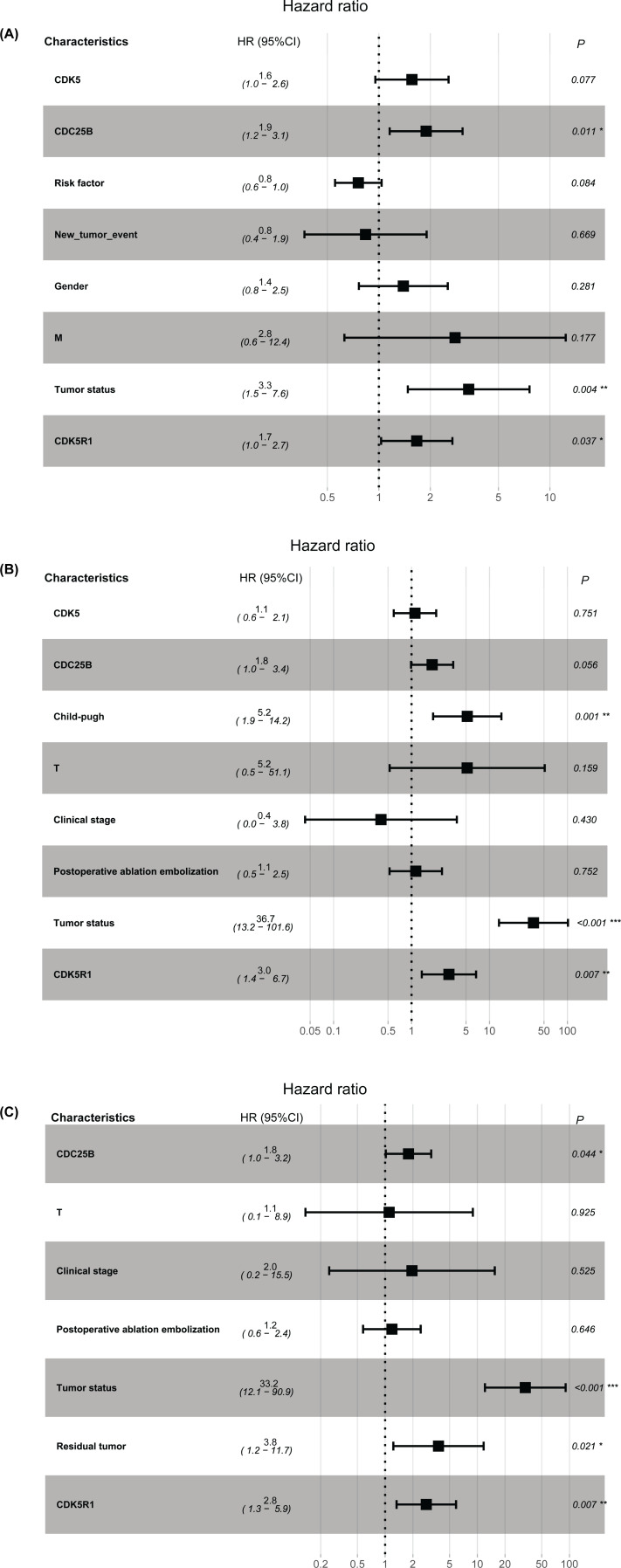
Association between clinicopathologic characteristics and survival outcome of HCC patient through univariate and multivariate Cox regression analysis Panel (**A**) showed CDK5R1 can independently predict adverse OS. Panel (**B**) indicated CDK5R1 can independently predict poor DFI. Panel (**C**) suggested that CDK5R1 can independently predict worse PFI; DFI, disease-free interval; OS, overall survival; PFI, progression-free interval.

Univariate analysis for OS with Cox regression model showed that poor OS had prominently correlation with CDK5R1 expression (high vs. low; *P*=0.033, HR = 2.0 (95% CI [1.1–3.9])), new tumor event (yes vs. no; *P*=0.012, HR = 3.0 (95% CI [1.3–7.0])), tumor status (with tumor vs. tumor free; *P*=0.001, HR = 4.0 (95% CI [1.8–9.2])), CDK5 expression (high vs. low; *P*=0.032, HR = 2.4 (95% CI [1.1–5.2])), CDC25B expression (high vs. low; *P*=0.005, HR = 3.1 (95% CI [1.4–6.8])) (Table 3). However, at multivariate Cox regression analysis, CDK5R1 expression (high vs. low; *P*=0.037, HR = 1.7 (95% CI [1.0–2.7])), tumor status (with tumor vs. tumor free; *P*=0.004, HR = 3.3 (95% CI [1.5-7.6])), the expression of CDC25B (high vs. low; *P*=0.011, HR = 1.9 (95% CI [1.2-3.1])) could independently predict adverse OS (Table 3, Figure 4A). Besides, this revealed that patients with elevated CDK5R1 have a 1.7 times higher risk of adverse OS than patients with low CDK5R1 expression.

**Table 3:** Association between clinicopathologic characteristics and HCC patient OS through univariate and multivariate analysis with Cox regression survival model

Characteristics	Univariate analysis			Multivariate analysis		
	HR	95%CI	*P*-value	HR	95%CI	*P*-value
Child-Pugh (B-C vs. A)	1.3	0.4-4.2	0.715			
Risk factor (Alcohol consumption and/or viral hepatitis vs. neither)	0.6	0.4-1.0	0.064	0.8	0.6-1.0	0.084
AFP (AFP≥400/20≤AFP<400 vs. AFP<20)	1.0	0.7-1.6	0.961			
New tumor event (yes vs. no)	3.0	1.3-7.0	0.012	0.8	0.4-1.9	0.669
Age (>40 vs. ≤40)	4.7	0.6-34.8	0.133			
Gender (male vs. female)	0.5	0.2-1.1	0.086	1.4	0.8-2.5	0.281
Histologic grade (G3-4 vs. G1-2)	1.6	0.7-3.3	0.247			
M (M1 vs. M0)	5.4	0.7-40.7	0.099	2.8	0.6-12.4	0.177
N (N1 vs. N0)	4.3	0.6-31.7	0.157			
T (T3-4 vs. T1-2)	1.2	0.5-3.0	0.691			
Clinical stage (III-IV vs. I-II)	1.4	0.6-3.3	0.454			
Postoperative ablation embolization (yes vs. no)	1.1	0.4-3.2	0.870			
Radiation therapy (yes vs. no)	0.8	0.5-1.3	0.997			
Vascular invasion (yes vs. no)	1.3	0.6-2.9	0.560			
Tumor status (with tumor vs. tumor free)	4.0	1.8-9.2	0.001	3.3	1.5-7.6	0.004
Family history (yes vs. no)	1.8	0.9-3.7	0.116			
Residual tumor (R1-2 vs. R0)	1.4	0.2-10.2	0.761			
CDK5 (high vs. low)	2.4	1.1-5.2	0.032	1.6	1.0-2.6	0.077
CAPN2 (high vs. low)	1.4	0.6-2.9	0.426			
CAPN1 (high vs. low)	1.6	0.7-3.3	0.242			
MAPT (high vs. low)	1.3	0.6-2.8	0.447			
CDC25B (high vs. low)	3.1	1.4-6.8	0.005	1.9	1.2-3.1	0.011
PAK1 (high vs. low)	1.5	0.7-3.0	0.310			
PPP1R1B (high vs. low)	1.0	0.5-2.2	0.947			
NTRK2 (high vs. low)	0.7	0.3-1.5	0.404			
NDEL1 (high vs. low)	0.9	0.4-2.0	0.829			
YWHAE (high vs. low)	0.9	0.4-1.9	0.840			
CDK5R1 (high vs. low)	2.0	1.1-3.9	0.033	1.7	1.0-2.7	0.037

Abbreviations: CI, confidence interval; HR, hazard ratio; M, distant metastasis; N, lymph node metastasis; OS, overall survival; T, topography distribution.

Univariate Cox analysis of DFI disclosed that highly expressed CDK5R1 had a prominent effect on DFI (*P*=0.000, hazard ratio [HR]= 2.7 (95% CI [1.6–4.5])), other clinical factor, for instance TNM (T (T3-4 vs. T1-2): *P*=0.043, HR = 2.0 (95% CI [1.0–3.9])), clinical stage (III-IV vs. I-II) (*P*=0.022, hazard ratio [HR]= 2.1 (95% CI [1.1–4.1])) was also associated with shorter DFI. At multivariate analysis, CDK5R1 (high vs. low; *P*=0.007, hazard ratio [HR]= 3.0 (95% CI [1.4-6.7])) were the clinicopathologic characteristics that remained significantly correlated with DFI (Table 4, Figure 4B). This showed that patients with increased CDK5R1 have a 3.0 times higher risk of poor DFI than patients with low CDK5R1 expression.

**Table 4:** Association between clinicopathologic characteristics and HCC patient DFI through univariate and multivariate analysis with Cox regression survival model

Characteristics	Univariate analysis			Multivariate analysis		
	HR	95%CI	*P*-value	HR	95%CI	*P*-value
Child-Pugh (B-C vs. A)	2.1	0.9–4.9	0.096	5.2	1.9-14.2	0.001
Risk factor (Alcohol consumption and/or viral hepatitis vs. neither)	0.9	0.6–1.2	0.341			
AFP (AFP≥400/20≤AFP<400 vs. AFP<20)	0.9	0.7–1.3	0.632			
New tumor event (yes vs. no)	2.4	1.4–4.0	0.996			
Age (>40 vs.≤40)	1.0	0.4–2.3	0.926			
Gender (male vs. female)	0.9	0.5–1.6	0.599			
Histologic grade (G3-4 vs. G1-2)	1.2	0.7–2.1	0.486			
M (M1 vs. M0)	0.8	0.6–1.1	1.000			
N (N1 vs. N0)	4.2	0.6–31.4	0.159			
T (T3-4 vs. T1-2)	2.0	1.0–3.9	0.043	5.2	0.5-51.1	0.159
Clinical stage (III-IV vs. I-II)	2.1	1.1–4.1	0.022	0.4	0.0-3.8	0.430
Postoperative ablation embolization (yes vs. no)	2.7	1.4–5.5	0.005	1.1	0.5-2.5	0.752
Radiation therapy (yes vs. no)	1.5	0.2–11.1	0.683			
Vascular invasion (yes vs. no)	1.0	0.5–2.0	0.910			
Tumor status (with tumor vs. tumor free)	35	13.4–93.5	0.000	36.7	13.2-101.6	0.000
Family history (yes vs. no)	1.1	0.6–2.1	0.639			
residual tumor (R1-2 vs. R0)	1.7	1.0–2.6	0.393			
CDK5 (high vs. low)	1.6	0.9–2.9	0.082	1.1	0.6-2.1	0.751
CAPN2 (high vs. low)	0.8	0.5–1.4	0.506			
CAPN1 (high vs. low)	1.5	0.8–2.5	0.178			
MAPT (high vs. low)	1.2	0.7–2.1	0.546			
CDC25B (high vs. low)	2.1	1.2–3.7	0.007	1.8	1.0-3.4	0.056
PAK1 (high vs. low)	1.1	0.6–2.0	0.676			
PPP1R1B (high vs. low)	0.8	0.5–1.5	0.537			
NTRK2 (high vs. low)	0.9	0.5–1.5	0.567			
NDEL1 (high vs. low)	0.9	0.5–1.6	0.682			
YWHAE (high vs. low)	1.0	0.6–1.7	0.987			
CDK5R1 (high vs. low)	2.7	1.6–4.5	0.000	3.0	1.4-6.7	0.007

Abbreviations: CI, confidence interval; DFI, disease-free interval; HR, hazard ratio; M, distant metastasis; N, lymph node metastasis; T, topography distribution.

Univariate Cox regression analysis of progression free interval (PFI) revealed that worse PFI was significantly associated with advanced TNM (T (T3-4 vs. T1-2): *P*=0.047, hazard ratio [HR]= 1.9 (95% CI [1.0–3.6])), clinical stage (III-IV vs. I-II) (*P*=0.026, HR = 2.0 (95% CI [1.1–3.8])), postoperative ablation embolization (yes vs. no) (*P*=0.003, HR = 2.7 (95% CI [1.4–5.1])), tumor status (with tumor vs. tumor free) (*P*=0.000, HR = 37.1 (95% CI [14.2–97.4])), residual tumor (R1-2 vs. R0) (*P*=0.001, HR = 6.4 (95% CI [2.2–18.3])), elevated CDK5R1 (*P*=0.000, HR = 2.5 (95% CI [1.5–4.2])), as shown in Table 4. Whereafter, multivariate analysis with Cox regression model uncovered that high expression of CDK5R1 was an independent prognostic factor for PFI, with an HR of 2.8 (*P*=0.007, 95% CI [1.3–5.9]), other clinical factor, for instance, the expression of CDC25B (high vs. low) (*P*=0.044, HR = 1.8 (95% CI [1.0–3.2])) was also independently associated with poor PFI (Table 5, Figure 4C). This uncovered that patients with highly expressed CDK5R1 have a 2.8 times higher risk of poor PFI than patients with low CDK5R1 expression.

**Table 5:** Association between clinicopathologic characteristics and HCC patient PFI through univariate and multivariate analysis with Cox regression survival model

Characteristics	Univariate analysis			Multivariate analysis		
	HR	95%CI	*P*-value	HR	95%CI	*P*-value
Child-Pugh (B-C vs. A)	1.8	0.8–4.3	0.164			
Risk factor (Alcohol consumption and/or viral hepatitis vs. neither)	0.8	0.6–1.1	0.244			
AFP (AFP≥400/20≤AFP<400 vs. AFP<20)	1.0	0.7–1.3	0.914			
New tumor event (yes vs. no)	3.4	2.1–5.7	0.995			
Age (>40 vs.≤40)	0.9	0.4–2.1	0.887			
Gender (male vs. female)	0.8	0.5–1.4	0.406			
Histologic grade (G3-4 vs. G1-2)	1.1	0.7–1.9	0.645			
M (M1 vs. M0)	5.0	0.7–37.4	0.116			
N (N1 vs. N0)	3.7	0.5–27.5	0.197			
T (T3-4 vs. T1-2)	1.9	1.0–3.6	0.047	1.1	0.1–8.9	0.925
Clinical stage (III-IV vs. I-II)	2.0	1.1–3.8	0.026	2.0	0.2–15.5	0.525
Postoperative ablation embolization (yes vs. no)	2.7	1.4–5.1	0.003	1.2	0.6–2.4	0.646
Radiation therapy (yes vs. no)	1.3	0.2–9.6	0.787			
Vascular invasion (yes vs. no)	1.2	0.7–2.1	0.590			
Tumor status (with tumor vs. tumor free)	37.1	14.2–97.4	0.000	33.2	12.1–90.9	0.000
Family history (yes vs. no)	1.1	0.6–1.9	0.785			
residual tumor (R1-2 vs. R0)	6.4	2.2–18.3	0.001	3.8	1.2–11.7	0.021
CDK5 (high vs. low)	1.5	0.9–2.5	0.131			
CAPN2 (high vs. low)	1.0	0.6–1.7	0.978			
CAPN1 (high vs. low)	1.4	0.8–2.4	0.186			
MAPT (high vs. low)	1.1	0.7–1.9	0.665			
CDC25B (high vs. low)	2.1	1.2–3.5	0.007	1.8	1.0–3.2	0.044
PAK1 (high vs. low)	1.3	0.8–2.2	0.350			
PPP1R1B (high vs. low)	1.0	0.6–1.6	0.858			
NTRK2 (high vs. low)	0.9	0.6–1.6	0.796			
NDEL1 (high vs. low)	1.0	0.6–1.8	0.870			
YWHAE (high vs. low)	1.1	0.6–1.8	0.797			
CDK5R1 (high vs. low)	2.5	1.5–4.2	0.000	2.8	1.3–5.9	0.007

Abbreviations: CI, confidence interval; HR, hazard ratio; M, distant metastasis; N, lymph node metastasis; PFI, progression free interval; T, topography distribution.

### CDK5R1-related signaling pathway performed on GSEA

**Figure 5:**
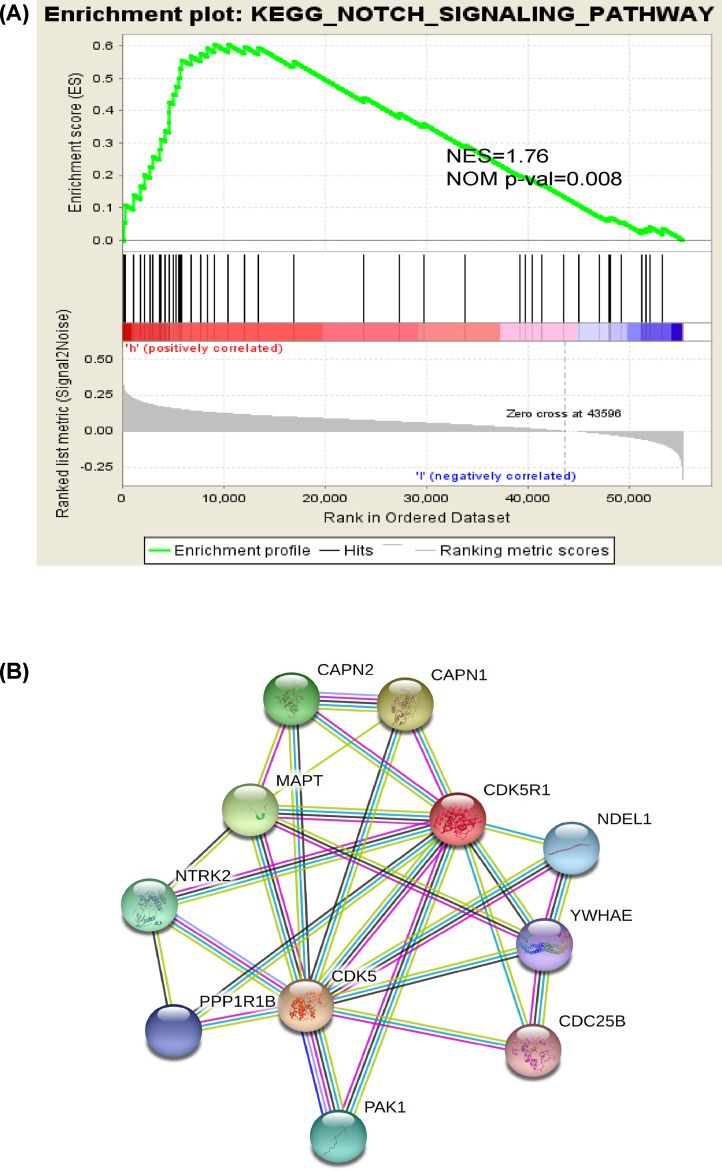
Enrichment plots from gene set enrichment analysis (GSEA) and PPI network of CDK5R1 (**A**) Results of GSEA showed notch signaling pathway were differentially enriched in high CDK5R1 expression phenotype. (**B**) PPI network of CDK5R1 suggested that CDK5R1 had close relationship with CDK5; ES, enrichment score; NES, normalized ES; FDR, false discovery rate; NOM *P*-val, normalized *P*-value; CDK5, cyclin-dependent kinase 5; CDK5R1, cyclin-dependent kinase 5 regulatory subunit 1; PPI, protein–protein interaction.

We employed Gene Set Enrichment Analysis (GSEA) to screen significantly activated signaling pathways between high and low CDK5R1 expression phenotype group, FDR <0.05 and NOM *P*-val < 0.05 indicated significant differences in enrichment of MSigDB collection (c2.cp.kegg.v7.0.symbols). In our analysis, 2 signaling pathways that were prominently enriched in high CDK5R1 expression phenotype were filtered out, including notch signaling pathway and non-small cell lung cancer. (Figure 5A, Table 6).

**Table 6:** Gene sets enriched in phenotype high.

MSigDB collection	Gene set name	NES	NOM *P*-val	FDR *q*-val
c2.cp.kegg.	KEGG_NOTCH_SIGNALING_PATHWAY	1.76	0.008	1.000
v7.0.symbols. gmt [Curated]	KEGG_NON_SMALL_CELL_LUNG_CANCER	1.60	0.040	1.000

Abbreviations: FDR, false discovery rate; NES, normalized enrichment score; NOM, nominal. Gene sets with NOM *P*-val < 0.05 and FDR *q*-val < 0.05 are considered as significant.

### CDK5R1-associated PPI network

The CDK5R1-associated PPI network was established with 11 points, 27 edges and an average point degree of 4.91(Figure 5B). The PPI network showed that some genes had close relationship with CDK5R1, for instance, CDK5, CAPN2, CAPN1, MAPT, CDC25B, PAK1, PPP1R1B, NTRK2, NDEL1 and YWHAE.

## Discussion

Despite considerable progress has been achieved in recent years, the morbidity and mortality of HCC are still increasing. Effective prediction of prognosis is of great significance for improving the survival of patients with HCC. However, so far, the prognostic biomarker has been limited. Although accumulative studies have demonstrated the clinical significance of CDK5R1 in various cancer types, up to date, the effect of CDK5R1 on HCC has not been reported. Therefore, a better understanding of the role of CDK5R1 in HCC and its potential prognostic value, as well as molecular mechanisms underlying its effects are required.

Up to date, there have been no reports on the role of CDK5R1 in HCC, but to our knowledge, CDK5R1 encodes the activator p35 of CDK5, which must be combined with the activator to work and thus CDK5R1 plays a pivotal role in regulating the appropriate activity of CDK5 [8]. That is to say, CDK5R1 has a close relationship with CDK5, which is consistent with our results in PPI network. Many studies have demonstrated that elevated CDK5R1 (p35) promotes the overexpression and activation of CDK5, which in turn promotes the initiation, progression, and metastasis of various tumors [11–15]. Accumulative studies have been proved that CDK5 is overexpressed and activated in HCC, and its excessive activation promotes the initiation and progression of HCC. Inhibition of CDK5 can increase the sensitivity of HCC cells to DNA-damaging agents and improve the responsiveness of patients with advanced HCC to sorafenib [19,20]; CDK5 knockout can inhibit the proliferation and promote apoptosis of HCC cells [21]. Taken together, we speculated that CDK5R1 may also play a critical role in the initiation, progression and metastasis of HCC.

In the present study, high-throughput RNA-seq data provided evidence that CDK5R1 was overexpressed in HCC tissues and an elevated expression of CDK5R1 had a close relationship with worse histologic grade, advanced clinical stage, poorer TNM, new tumor event, higher serum AFP value as well as shorter survival time. These suggested that there may be a high probability of HCC recurrence, invasion and metastasis in patients with elevated CDK5R1, and highly expressed CDK5R1 may herald poor prognosis. Further, univariate and multivariate Cox regression analysis disclosed that under the influence of excluding other clinicopathological factors such as genes closely related to CDK5R1, CDK5R1 was still the factor that can independently predict poor OS, DFI and PFI. Although the gene CDC25B, which is strongly associated with CDK5R1, may also independently predict poor OS and PFI.

We further investigated the function of CDK5R1 and the probable mechanism underlying the effects of CDK5R1 on the progression and metastasis HCC based on GSEA. GSEA has wide applicability and is one of the most commonly used approaches for path enrichment analysis. Compared with traditional pathway enrichment analysis such as gene ontology (GO) and Kyoto Gene and Genome Encyclopedia (KEGG), GSEA can detect the expression changes of gene sets rather than individual genes, and GSEA can detect subtle enrichment signals, which makes the results more reliable and flexible [16]; However, GSEA’s functional class scoring (FCS) approach has some limitations. When FCS analyses each pathway, it is likely to treat genes with different fold changes equally, although some genes with larger fold changes should receive greater weight, which may overlook the biological significance of certain genes and their complex interconnections. In addition, some pathway annotation information is insufficient, which makes it difficult to set the appropriate threshold to determine the gene set. Some genes also have insufficient annotation information, which reduces the sensitivity of GSEA detection [22]. As there is little literature on CDK5R1, with the performance of GSEA, we only found that notch signaling pathway and non-small cell lung cancer were significantly enriched in the CDK5R1 high expression phenotype.

Cancer stem cell is the origin of tumor, it promotes the growth and development of tumor cells and is an important cause of tumor recurrence [23–25]. In addition, cancer stem cells resist chemotherapy and radiation and are difficult to eradicate, which can lead to recurrence and metastasis for years after therapeutic treatment [26]. Studies have shown that tumor stem cells can make patients more susceptible to recurrence after HCC surgical resection [27]. The notch signaling pathway is one of the pivotal pathways that regulate the differentiation and development of cancer stem cells. It plays a key role in the self-renewal and angiogenesis of cancer stem cells. The abnormal notch signaling pathway as a carcinogen is closely linked to the occurrence, progression, and metastasis of a variety of cancers [28]. Notch signaling pathway blockers can delay the generation of tumors and effectively reduce the occurrence of tumors and self-renewal of cancer stem cells, which is expected to cure tumors by completely removing cancer stem cells [23]. Vitro experiments show that vascular endothelial CDK5 inhibitors can influence the migration and proliferation of vascular endothelial cells by inhibiting NOTCH-driven angiogenesis, thereby affecting tumor angiogenesis and ultimately inhibiting tumor growth [29]. Previous studies have also reported that DAPT, a Notch inhibitor in the nervous system, can down-regulate CDK5 activity [30]. In summary, CDK5R1 may participate in the progression and migration of HCC by regulating the notch signaling pathway. The present study is the first to report the role of CDK5R1 in HCC and the regulatory effect of CDK5R1 on the notch signaling pathway in HCC.

Although our current study has improved our understanding of the role of CDK5R1 in HCC, there are still some limitations. First, the sample size of cancer patients in the TCGA database was significantly higher than that of the control patients. Second, the absence of clinical factors in the public database, such as specific details of the patient’s medication and/or surgical treatment, also affects the patient’s prognosis. Third, the protein level of CDK5R1 in HCC and its direct role in HCC progression and metastasis remain to be further validated *in vitro*. Fourth, due to the limitations of GSEA, and so far, too little research has been done on CDK5R1, other important signaling pathways regulated by CDK5R1 may be missed. Finally, the present study is a retrospective study, and prospective studies should be conducted in the future to make up for the limitations of the retrospective study. Although the present study has some limitations, it does provide clues for studying the function of CDK5R1 in HCC, and provides targets and potential prognostic markers for the treatment of HCC.

## Conclusion

Patients with elevated CDK5R1 may have a poor prognosis, increased CDK5R1 may act as a promising independent prognostic marker of poor survival and therapeutic target in HCC. Besides, it may participate in the progression and migration of HCC through regulating the notch signaling pathway.

## Data Availability

The datasets generated and/or analyzed during the current study are available in the TCGA repository, https://portal.gdc.cancer.gov/repository?facetTab=cases; and GEO repository https://www.ncbi.nlm.nih.gov/geo/query/acc.cgi?acc=GSE121248 and https://www.ncbi.nlm.nih.gov/geo/query/acc.cgi?acc=GSE62232.

## Abbreviations

CDK5: cyclin-dependent kinase 5
CDK5R1: cyclin-dependent kinase 5 regulatory subunit 1
DFI: disease-free interval
DSS: disease-specific survival
GEO: Gene Expression Omnibus
GSEA: Gene Set Enrichment Analysis
OS: overall survival
PFI: progression-free interval
